# Silibinin Triggers Mitochondrial Apoptosis and Declines Clonogenic Potential in Detroit 562 Human Pharyngeal Carcinoma Cells

**DOI:** 10.3390/medicina61122197

**Published:** 2025-12-11

**Authors:** Serban Talpos, Doina Chioran, George Cătălin Alexandru, Ștefania Dinu, Elena-Dorina Coricovac, Andreea Smeu, Diana Haj Ali, Camelia Szuhanek, Malina Popa

**Affiliations:** 1 Discipline of Oral and Maxillo-Facial Surgery, Faculty of Dental Medicine, “Victor Babes” University of Medicine and Pharmacy Timisoara, Revolutiei Boulevard 9, 300041 Timisoara, Romania; 2 Department of Anesthesiology and Oral Surgery, “Victor Babes” University of Medicine and Pharmacy Timisoara, Eftimie Murgu Sq. No. 2, 300041 Timisoara, Romania; 3 Doctoral School, “Victor Babes” University of Medicine and Pharmacy Timisoara, Eftimie Murgu Square, No.2, 300041 Timisoara, Romania; 4 Faculty of Medicine, “Victor Babes” University of Medicine and Pharmacy Timisoara, Eftimie Murgu Square, No.2, 300041 Timisoara, Romania; 5 Department of Pedodontics, Faculty of Dental Medicine, “Victor Babes” University of Medicine and Pharmacy Timisoara, 9 No., Revolutiei Bv., 300041 Timisoara, Romania; popa.malina@umft.ro; 6 Pediatric Dentistry Research Center, Faculty of Dental Medicine, “Victor Babes” University of Medicine and Pharmacy Timisoara, 9 No., Revolutiei Bv., 300041 Timisoara, Romania; 7 Department of Toxicology, Drug Industry, Management and Legislation, Faculty of Pharmacy, “Victor Babes” University of Medicine and Pharmacy Timisoara, 2 Eftimie Murgu Square, 300041 Timisoara, Romania; dorinacoricovac@umft.ro (E.-D.C.);; 8 Research Center for Pharmaco-Toxicological Evaluations, “Victor Babes” University of Medicine and Pharmacy Timisoara, 2 Eftimie Murgu Square, 300041 Timisoara, Romania; 9 Orthodontic Research Center ORTHO-CENTER, Discipline of Orthodontics I, Faculty of Dental Medicine, “Victor Babes” University of Medicine and Pharmacy Timisoara, 9 No., Revolutiei Bv., 300041 Timisoara, Romania; cameliaszuhanek@umft.ro

**Keywords:** silibinin, oropharyngeal cancer, human papillomavirus, gingival fibroblasts, alternative treatment, mitochondrial apoptosis

## Abstract

*Background and Objectives*: Oropharyngeal squamous cell carcinoma (OPSCC) is a common type of head and neck cancer with a progressive incidence in recent years. The limitations and the side effects associated with the current treatments require new therapeutic alternatives. Silibinin (SIL) is a phytocompound with multifaceted properties that has demonstrated antitumor effects in several types of cancer. The aim of this study was to assess the potential anticancer effects of SIL in Detroit 562 human pharyngeal cancer cells, an ideal model for HPV-negative OPSCC. *Materials and Methods*: Detroit 562 cells and HGF-1- human gingival fibroblasts were used as experimental models. For the mechanistic investigations, different methods, such as MTT assay, bright field microscopy, immunofluorescence staining, and specific assays and kits were applied to quantify intracellular ROS production, activation of caspases, and the colony formation assay. *Results*: Treatment with SIL (25–200 µM) for 48 h induced a selective cytotoxic effect in Detroit 562 cancer cells, being minimally toxic to healthy cells. The cytotoxic mechanism of action was characterized by a decreased cell viability, morphological alterations, elevation of intracellular ROS, decreased mitochondrial potential, mitochondrial and nuclear dysmorphologies, activation of caspases 9 and 3/7 and apoptosis occurrence, and decreased long-term colony formation. *Conclusions*: These findings show that SIL could represent a potential alternative therapy for HPV-negative OPSCC by triggering mitochondrial apoptosis and exerting a decline in the colonogenicity of Detroit 562 cancer cells.

## 1. Introduction

Oropharyngeal squamous cell carcinoma (OPSCC), also known as throat cancer or tonsil cancer, is a form of head and neck cancer [1], which is ranked as the seventh most common cancer globally [2], and represents the cause of death for approximately 300,000 patients annually [3]. OPSCC is a condition that affects the tongue, the tonsils, the soft palate, and the posterior and lateral pharyngeal walls, and in 90% of cases, these cancers are diagnosed as squamous cell cancers [1]. In recent years, OPSCC has become a public health challenge due to several factors, such as (i) an increased incidence in both old and young people [4], (ii) the intricate interplay between oral mucosal cells, oral microbiome and tumor microenvironment that leads to the development and progression of OPSCC [5], (iii) late diagnosis since early stage OPSCC is asymptomatic and the oropharynx is an anatomic area with difficult access [6], (iv) resistance of cancer cells to conventional therapy, poor prognosis and a high rate of relapse, and (v) the multiple side-effects of the existent treatment [7]. In 2017, the World Health Organization (WHO) Classification of Head and Neck Tumors (“Blue Book”) grouped the OPSCC into HPV-positive (mainly in young people—an increased incidence in developed countries) and HPV-negative OPSCC (diagnosed in older people with smoking and alcohol consumption habits) [8]. These two entities present unique clinicopathological and epidemiological features that impose different therapy approaches, especially since, according to the literature, patients with HPV-positive forms showed a better response to conventional therapies and a higher survival rate as compared to HPV-negative ones [9]. Even though the records for HPV-positive OPSCC treatment strategies have been rather considerable in recent years, for the HPV-negative type, there is limited evidence for the best treatment option [10], a situation that must be solved in the near future.

The heterogeneity of OPSCC requires a multifaceted management that involves collaboration among healthcare professionals (oncologist, surgeon, histopathologist, radiation oncologist, otorhinolaryngologist, dentist, psychotherapist, dietitian, clinical speech pathologist, dental hygienist, and a chemotherapy nurse) to obtain better outcomes and to improve the patient’s quality of life [2,11,12]. Although the substantial progress recorded in terms of OPSCC treatment options, including chemotherapy, radiation, surgery (as conventional approaches), immunotherapy [13], and targeted therapy (as current therapy), as well as gene therapy, epigenetic modulators, and nanomedicine-based and PROTAC-based treatments (as emerging approaches), the survival rate is still low (around 50%) [7]. Therefore, finding new, safer alternatives, like the use of natural compounds as adjuvant treatment, has become mandatory.

The use of natural compounds in cancer treatment has gained substantial attention in recent decades, including for the treatment and prevention of oral cancer. Plant-based compounds offer the benefit of being selective on tumor cells, with very little or no toxicity on healthy cells, a feature that makes them ideal therapeutic alternatives [14]. Silibinin (SIL), a flavonolignan, is a derivative of *Silybum marianum* and the main component in silymarin. SIL proved a broad spectrum of biological effects, including anti-inflammatory, antioxidant, antineoplastic and hepatoprotective effects [15]. Previous studies reported pleiotropic antineoplastic effects of SIL in different types of cancer (breast, bladder, prostate, pancreatic, thyroid, cervical, ovarian, skin, esophageal and salivary gland cancers, hepatocellular carcinoma, and renal cell carcinoma) by exerting a multifaceted mechanism of action [15,16]. In addition, past research showed that SIL displayed anti-migratory and anti-invasive capacity on tongue cancer cells [17], triggered apoptosis via mitochondrial pathway in human oral squamous cell carcinoma cells [18] and reduced the side effects of radiotherapy when used concomitantly [19]. Still, reports regarding the effects of SIL on cancer cells of pharyngeal origin are rather lacking.

Because several recent studies conducted in different parts of Romania (Timisoara and Iasi) revealed that the incidence of HPV-negative OPSCC is higher as compared to the HPV-positive form [20,21], this study offers a thorough in vitro toxicological evaluation of SIL effects on Detroit 562 cancer cells (cells of pharyngeal origin), an ideal in vitro model for investigating alternative treatment options for HPV-negative OPSCC.

The objective of this study was to verify the hypothesis that SIL displays anticancer effects in Detroit 562 pharyngeal cancer cells through the induction of mitochondria-induced apoptosis and antiproliferative activity. In this regard, cytotoxicity was assessed by measuring cell viability, morphological changes, mitochondrial apoptosis, and clonogenic survival. Furthermore, SIL’s selectivity was evaluated by comparing its effects on cancer cells to those on healthy HGF-1 fibroblasts. The findings provide insight into the mechanisms behind SIL’s selective anticancer activity.

## 2. Materials and Methods

### 2.1. Reagents

Trypsin-EDTA solution, phosphate-buffered saline (PBS), dimethyl sulfoxide (DMSO), specific cell culture media (EMEM and DMEM), penicillin/streptomycin, and fetal bovine serum (FBS) were obtained from ATCC (Manassas, VA, USA). The MTT reagent (3-(4,5-dimethylthiazol-2-yl)-2,5-diphenyltetrazolium bromide) was purchased from Roche Holding AG (Basel, Switzerland). Hoechst 33342 nuclear stain and MitoTracker Red CMXRos were supplied by Thermo Fisher Scientific (Waltham, MA, USA) and the JC-1 Mitochondrial Membrane Potential Assay Kit by Elabscience (Houston, TX, USA). Silibinin, Acridine Orange, Propidium Iodide and Sodium lauryl sulfate (SLS) were obtained from Sigma-Aldrich (Merck KGaA, Darmstadt, Germany). The 4% paraformaldehyde in PBS was bought from Santa Cruz Biotechnology (Dallas, TX, USA). Crystal violet 1% solution was procured from Electron Microscopy Sciences (Hatfield, PA, USA), and the Caspase-Glo 3/7, Caspase-Glo 9, and ROS-Glo H_2_O_2_ assay kits were obtained from Promega Corporation (Madison, WI, USA). All the chemicals used in the study were of analytical purity and suitable for cell culture conditions.

### 2.2. Cell Lines and Cell Culture Conditions

This study was carried out using two cell lines of human origin obtained from the American Type Culture Collection (ATCC, Manassas, VA, USA): Detroit 562 (CCL-138™, human pharyngeal carcinoma cell line) and HGF-1 (CRL-2014™, normal human gingival fibroblasts). For culture and growth, Detroit 562 cells required EMEM medium (ATCC^®^ 30-2003™) supplemented with 10% fetal bovine serum (FBS) and 1% antibiotic mixture (100 U/mL penicillin and 100 μg/mL streptomycin), whereas the HGF-1 cells were cultured in DMEM medium (ATCC^®^ 30-2002™) with the same supplementation (10% FBS and 1% penicillin-streptomycin). Throughout the experiment, both cell lines were incubated at 37 °C in a humidified atmosphere containing 5% CO_2_.

### 2.3. Instruments

The data recordings and the microscopic image acquisition were performed using a Cytation 5 (multimode plate reader) and a Lionheart FX Automated Microscope, respectively, supplied by BioTek Instruments (Winooski, VT, USA). Data analysis was conducted by the means of Gen5 Microplate Data Collection and Analysis Software (Version 3.14; BioTek Instruments, USA).

### 2.4. Cell Viability Evaluation Using MTT Colorimetric Assay

Cell viability was determined using the MTT assay. For cell viability assessment, a number of 1 × 10^4^ cells /well (Detroit 562 and HGF-1 cells) were plated in complete growth medium in 96-well plates to reach the desired confluence. The cells were further treated with increasing concentrations of SIL (25, 50, 75 100, and 200 µM) for 48 h. SIL was solubilized in DMSO obtaining a stock solution of 100 mM. After the 48 h of treatment, the culture medium was replaced with a fresh one and 10 µL of MTT was added. The 3 h incubation step was followed by the addition of 100 µL MTT solubilizing solution to each well, and the plate was kept for 30 min at room temperature and dark. Finally, the absorbance was measured at 570 and 630 nm. These experimental conditions were in accordance with the protocol described by Iliescu et al. [22].

### 2.5. Cellular Morphology Assessment

The modifications/alterations of cellular morphology in Detroit 562 cells following a 48 h treatment with SIL (25, 50, 75, 100, and 200 µM) were monitored by taking representative images under brightfield illumination (Lionheart FX automated microscope) of the control (untreated) and treated cells. The obtained pictures were processed using the Gen5™ Microplate Data Collection and Analysis Software (Version 3.14) from BioTek Instruments Inc. (Winooski, VT, USA).

### 2.6. Detection of Intracellular Reactive Oxygen Species (ROS)

For the measurement of intracellular ROS generation in Detroit 562 cells, the ROS-Glo™ H_2_O_2_ Assay (Promega, USA) was applied. The experimental protocol was conducted according to manufacturer’s recommendations and adapted to our laboratory conditions. A number of 1 × 10^4^ Detroit 562 cells/well were grown in 96-well opaque white plates. The cells were treated with different concentrations (25, 50, 75, 100, and 200 µM) of SIL for 48 h. After 42 h of exposure, 20 μL of ROS-Glo™ H_2_O_2_ substrate solution, prepared according to the manufacturer’s protocol, were added to each well. The plates were then returned to the incubator and maintained under standard culture conditions (37 °C, 5% CO_2_ atmosphere) until the completion of the 48 h treatment period. Subsequently, 100 μL of ROS-Glo™ detection solution was added to each well, and the plates were incubated for 20 min at room temperature in the dark. Luminescence was recorded using Cytation 5 multimode Reader (BioTek Instruments, USA). The luminescence data were processed following the method described by Yonbawi et al. [23]. The values were normalized to the control cells (untreated), and the results were expressed as the percentage of ROS reduction in each treatment group relative to the control (100%).

### 2.7. Mitochondrial Membrane Potential (ΔΨm) Assay—JC-1 Staining

To verify the potential impact of SIL treatment on Detroit 562 cells’ mitochondrial membrane potential, the fluorescent probe JC-1 assay was performed according to the manufacturer’s specifications. The cells (1 × 10^4^ cells/well) were seeded in a black walled 96 flat-bottom clear plate, and after the confluence reached approximately 70%, the cells were treated with SIL (25, 50, 75, 100, and 200 µM) for 48 h. Next, the medium was removed, and the cells were gently washed with PBS. Subsequently, they were incubated with JC-1 dye (5 µM; 100 μL per well) for 45 min at 37 °C. Following staining, the cells were rinsed twice with PBS to remove the excess dye, and the fluorescence imaging was performed using a Lionheart FX automated microscope. The processing of the images was done by the means of the Gen5™ Microplate Data Collection and Analysis Software (Version 3.14). The red and green fluorescence intensities were recorded at 590 nm and 529 nm, respectively, using a Cytation 5 multimode reader (BioTek Instruments, USA). The ΔΨm was calculated by determining the ratio of JC-1 aggregate (red) to monomeric (green) fluorescence. The obtained ratio values were then normalized to the control group (set to 100%), and the results were expressed as percentage relative to control (100%) [24].

### 2.8. Mitochondria and Nuclei Immunofluorescence Staining

To assess the mitochondrial and nuclear morphology, Detroit 562 cells (1 × 10^5^ cells/well) were cultured in 12-well plates and treated for 48 h with SIL (25, 50, 75, 100, and 200 µM) when the optimal confluence was achieved. The MitoTracker dye stock solution (1 mM) in DMSO was diluted in the complete culture medium to a final concentration of 300 nM. Following a 30 min incubation with the staining solution under standard culture conditions, the cells were washed with the culture medium to remove the excess dye. After MitoTracker staining, cells were fixed with 4% paraformaldehyde for 10 min at room temperature to preserve cellular structures, followed by thorough washing with PBS. To evaluate the nuclear alterations induced by SIL, Hoechst 33342 staining was performed. After fixation, the Hoechst 33,342 solution (diluted 1:2000 in PBS) was added to each well and incubated for 5–10 min in the dark. The staining solution was then removed, and the wells were washed three times with PBS. Representative images were captured using the Lionheart FX automated microscope at 20× magnification. Image analysis was carried out using Gen5™ Microplate Data Collection and Analysis Software (version 3.14; BioTek Instruments Inc., Winooski, VT, USA).

### 2.9. Caspase 3/7 and Caspase 9 Activation

To evaluate the effect of SIL on caspase-3/7 and caspase-9 activities, Detroit 562 cells were seeded in opaque white 96-well plates at a density of 10,000 cells per well. After cell attachment, the cells were treated with increasing concentrations (25, 50, 75, 100, and 200 µM) of SIL for 48 h. Following treatment, the plates were equilibrated to room temperature, and the culture medium was removed. Next, a volume of 100 µL of Caspase-Glo^®^ 3/7 or Caspase-Glo^®^ 9 reagents (Promega, USA), prepared according to the manufacturer’s protocol, were added in each well. The plates were gently mixed on an orbital shaker for 30 s to ensure homogeneity and incubated in the dark for 3 h at room temperature. Luminescence was recorded using a Cytation 5 multimode Reader (BioTek Instruments, USA). The luminescence values were processed according to the method described by Wen et al. [25]. The data were normalized to the control cells (untreated), and caspase activity in each group was expressed as a percentage relative to the control (100%).

### 2.10. Acridine Orange/Propidium Iodide (AO/PI) Assay

To confirm that SIL induced apoptosis in Detroit 562 cells, the double staining AO/PI assay was performed. After 48 h of exposure to SIL (25, 50, 75, 100 and 200 µM), 100 µL of a solution prepared in the Detroit 562 cells’ complete growth medium including a 10% mixture of 10 µg/mL AO and 10 µg/mL PI, were added to the cells (10^4^ cells/well) cultured in 96-well plates. Subsequently the plates were kept for 10 min at room temperature under dark conditions. In the final step, images were recorded using a Lionheart FX automated microscope and analyzed using Gen5 Microplate software Data Collection and Analysis Software. The experimental procedure was conducted in accordance with the protocol applied by Dahma et al. [26].

### 2.11. Colony Formation Assay

To assess the potential impact of SIL on the clonogenic capacity of Detroit 562 cells, a colony formation assay was performed according to the protocol described by Marcovici et al. [27]. The protocol consisted of the following steps: (i) seeding of cells (100 cells/well, in a 96 well-plate) and attachment to the plate, (ii) treatment with SIL (25, 50, 75, 100, and 200 µM) for 48 h, (iii) at the end of the 48 h treatment, the culture medium was replaced, and medium renewal was performed regularly throughout a 10 days incubation period, (iv) after the incubation period, the cells were fixed with 4% paraformaldehyde for 10 min at room temperature, washed twice with PBS, and subsequently stained with 0.2% crystal violet solution prepared in PBS for 10 min, (v) removal of the excess dye by washing twice with distilled water, (vi) image acquisition of the colonies using a Lionheart FX automated microscope, and (vii) cell lysis using 1% sodium lauryl sulfate (SLS) followed by absorbance readings at 550 nm using a Cytation 5 multimode reader. The images were analyzed using Gen5 Microplate software Data Collection and Analysis Software. The colony formation rate was determined according to the method described by Han et al. [28], calculating the ratio by dividing the absorbance values of each group to that of the control group.

### 2.12. Statistical Analysis

All assays were executed in three independent experiments, each performed in technical triplicates. The results are expressed as mean ± standard deviation (SD). The normality of data distribution was assessed using the Shapiro–Wilk test. As the data followed a normal distribution, statistical analysis was performed using one-way ANOVA followed by Dunnett’s multiple comparisons test. The analysis was conducted using GraphPad Prism version 10.2.3 (GraphPad Software, San Diego, CA, USA; www.graphpad.com). Data results that were statistically significant were marked with “*”: * *p* < 0.05; ** *p* < 0.01; *** *p* < 0.001; **** *p* < 0.0001.

## 3. Results

### 3.1. SIL Triggered a Cell-Type Dependent Cytotoxicity: Potent Effects in Detroit 562 Cancer Cells Versus Minimal Toxicity in HGF-1 Healthy Gingival Fibroblasts

**Figure 1:**
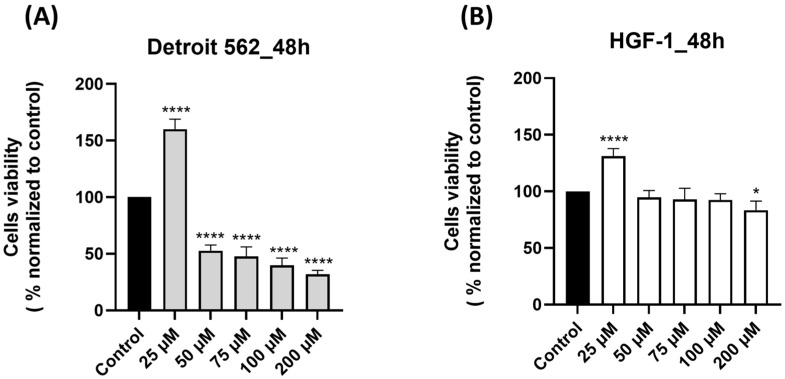
Graphical representations of the effect induced by silibinin—SIL (25–200 µM) treatment on cell viability after 48 h: (**A**) Detroit 562 pharyngeal cancer cells and (**B**) HGF-1 healthy gingival fibroblasts. The results are expressed as percentages normalized to control (untreated cells—100% viable) and are presented as mean values ± standard deviation. Statistical analysis and definitions of symbols are described in the Statistical analysis section. Data results that were statistically significant were marked with “*”: * *p* < 0.05 and **** *p* < 0.0001.

Prior to this study, we conducted a comprehensive search of the literature to verify the current state of knowledge regarding the impact of SIL on human pharyngeal cancer cells and we identified a paucity of in vitro studies in this direction. Based on this finding, we conducted an in vitro study to evaluate the biological response of the human pharyngeal cancer cell line Detroit 562 to SIL treatment. First, we assessed the effect of SIL (25, 50, 75, 100 and 200 µM) on Detroit 562 cells’ viability after a 48 h treatment by performing a MTT assay. To confirm the selectivity of SIL for cancer cells, we also verified its effect on healthy human gingival fibroblasts HGF-1 under the same experimental conditions. SIL treatment determined dose-dependent effects on Detroit 562 cells viability percentages, as follows: the lowest concentration tested—25 µM induced a significant increase in cells viability (160.15%), whereas at 50 µM and by increasing concentration (75–200 µm), a marked decrease was noticed and the lowest percentage of viability—31.80% was calculated for the highest concentration tested—200 µM (50 µM < 75 µM < 100 µM < 200 µM: 50.77% > 47.67% > 39.70%> 31.80%—Figure 1A). By comparison, HGF-1 cells viability (Figure 1B) was slightly decreased by the SIL treatment only at the highest concentration tested—200 µM (83.48%), whereas at 25 µM an increase in cell viability was observed (131.01%) and within the concentration range of 50–100 µM, the viability percentage values were similar to the control. These results indicate a selective cytotoxic effect of SIL in Detroit 562 cancer cells and a reduced toxicity in HGF-1 cells. The impact of DMSO (the solvent used for SIL solubilization) was also verified on both Detroit 562 and HGF-1 cells by applying the same experimental conditions, and the cell viability percentages obtained were comparable with control (untreated) cells; therefore, all the results were normalized to control cells.

### 3.2. SIL Altered the Cellular Morphology of Detroit 562 Cancer Cells

**Figure 2:**
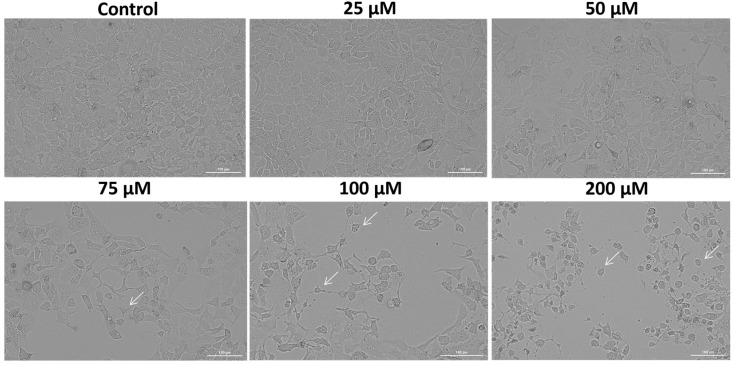
The morphological appearance of Detroit 562 cancer cells after 48 h treatment with silibinin (SIL)—25, 50, 75, 100 and 200 µM. The white arrows indicate morphological alterations. Images were obtained at 20× magnification, and the scale bar indicates 100 µm.

In order to validate the SIL-induced cytotoxicity on Detroit 562 cancer cells, a microscopic examination of cellular morphology was conducted following the 48 h treatment. The bright field microscopic analysis revealed a dose-dependent effect of SIL on Detroit 562 cells’ morphology and confluence (Figure 2). At the lowest concentration—25 µM, SIL had no toxic impact on cells morphology, the cells kept their adherent properties and their epithelial morphology, exhibiting an appearance similar to control (untreated) cells. Starting with 50 µM, slight morphological alterations were observed, including mild cell shrinkage and a reduction in confluence. Treatment with 75 and 100 µM led to more pronounced changes, characterized by the presence of rounded cells, loss of cell-to-cell contact and reduced confluence. At the highest concentration–200 µM, a marked deterioration of cellular morphology was evident, including extensive cell rounding, fragmentation, and substantial shrinkage, indicating severe cytotoxic effects (white arrows). These results are in agreement with the data obtained for cell viability determination.

### 3.3. SIL Stimulates Intracellular ROS Production in Detroit 562 Cancer Cells in a Dose-Dependent Manner

**Figure 3:**
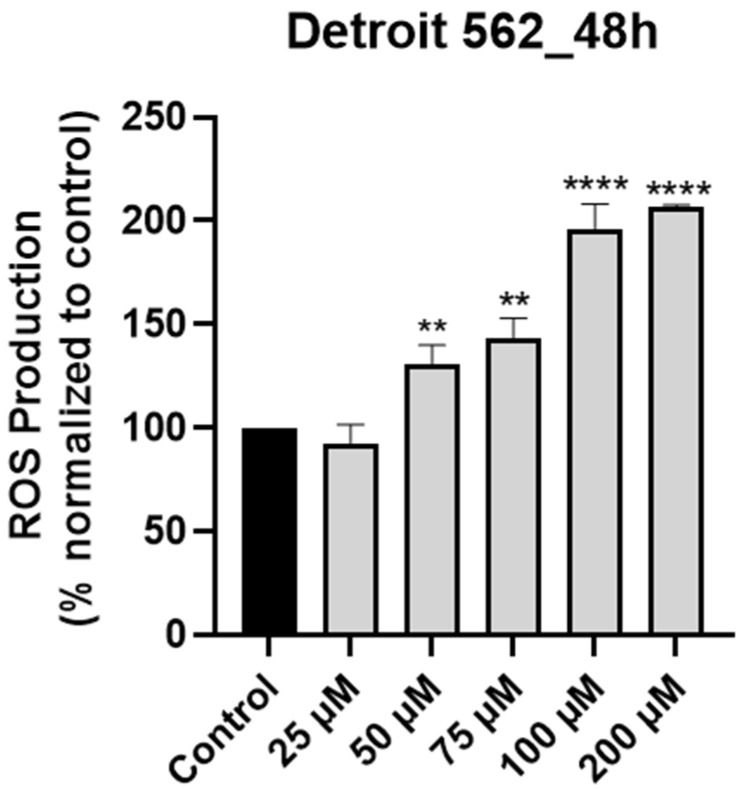
Graphical representation of the impact induced by silibinin (SIL—25, 50, 75, 100 and 200 µM) treatment on intracellular ROS production in Detroit 562 cells after 48 h. The results are expressed as percentages normalized to control and are presented as mean values ± standard deviation. Statistical analysis and definition of symbols are described in the Statistical analysis section. Data results that were statistically significant were marked with “*”: ** *p* < 0.01 and **** *p* < 0.0001.

According to the literature, SIL induced apoptosis in various cancer cell types through the intrinsic (mitochondrial) pathway, a process that was associated with increased intracellular levels of ROS [15]. Moreover, elevated levels of intracellular ROS are considered an initial event in the activation of the mitochondrial cascade of apoptosis [29]. Given the association between SIL and ROS in other models, we hypothesized that a similar mechanism occurs in Detroit 562 cells. We tested this by quantifying intracellular H_2_O_2_ production following SIL treatment using the ROS-Glo™ Assay (Promega, USA). Following treatment (48 h), SIL exerted a dose-dependent stimulatory effect on intracellular ROS production and the highest level (206.34%) was recorded at the highest concentration tested—200 µM (25 µM < 50 µM < 75 µM < 100 µM < 200 µM: 92.53% < 130.27% < 143.37% < 196.06% < 206.34%—Figure 3). The results were calculated by normalization to control (untreated) cells.

### 3.4. SIL-Elicited Mitochondrial Depolarization in Detroit 562 Cancer Cells

**Figure 4:**
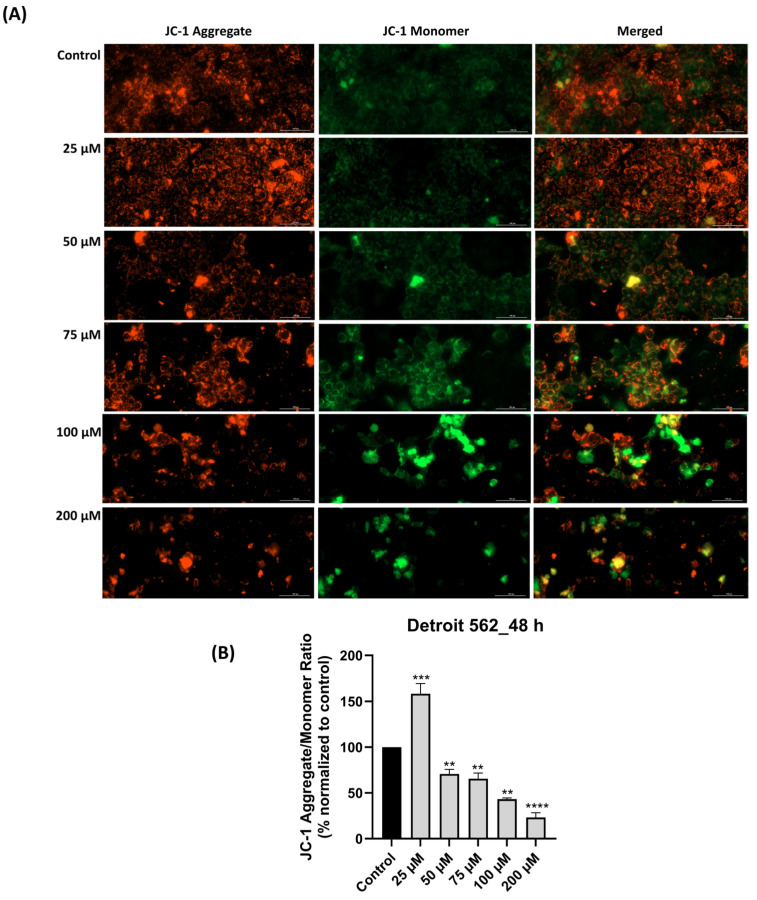
Mitochondrial membrane potential impairment following silibinin (SIL—25, 50, 75, 100 and 200 µM) treatment for 48 h: (**A**) JC-1 assay images (in red—JC-1 aggregate—intact mitochondria and in green—JC-1 monomer—damaged mitochondria). The images were taken at 20× magnification, and the scale bar was 100 µm. (**B**) Graphical representation of JC-1 aggregate/monomer ratio expressed as a percentage normalized to control. Results are presented as mean values ± standard deviation. Statistical analysis and definition of symbols are described in the Statistical analysis section. Data results that were statistically significant were marked with “*”: ** *p* < 0.01; *** *p* < 0.001; **** *p* < 0.0001.

Since elevated intracellular ROS levels are known to disrupt mitochondrial function [29], we proceeded to evaluate the integrity of the mitochondrial membrane potential via the JC-1 assay. The results, as illustrated in Figure 4A,B, showed dose-dependent changes in mitochondrial membrane potential after SIL treatment for 48 h. At 25 µM, a strong red fluorescence and an increased aggregate/monomer ratio (158.38%) suggested a boost in mitochondrial activity and mitochondrial integrity. Starting with 50 µM concentration (70.74%), the red signal gradually decreased while green fluorescence became more visible, indicating early depolarization of the mitochondrial membrane. These effects were more pronounced at 75 and 100 µM, where the aggregate/monomer ratio was 65.49% and 48.19%, respectively. At 200 µM, the green signal dominated (the aggregate/monomer ratio was 23.25%), pointing to a massive loss of mitochondrial potential.

### 3.5. SIL Induces Mitochondrial Damage and Nuclear Morphology Alterations Dose-Dependently

**Figure 5:**
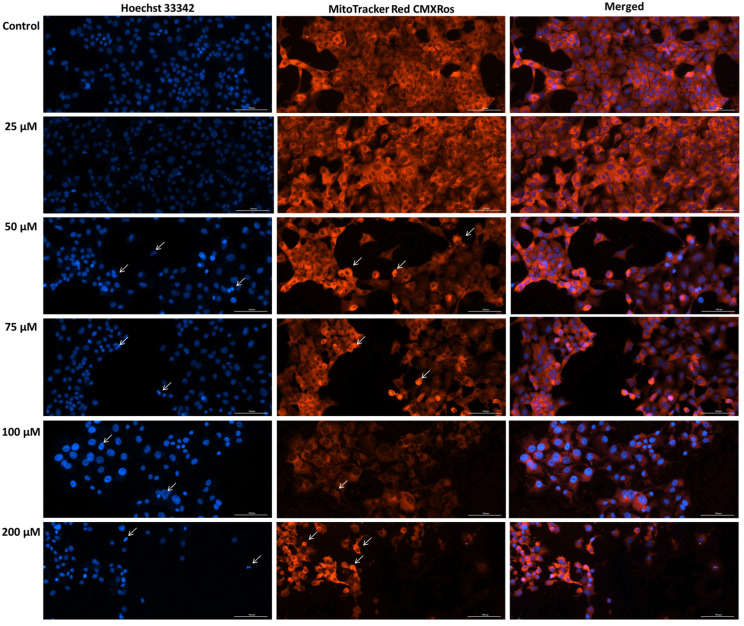
Representative images of the impact of silibinin (SIL-25, 50, 75, 100 and 200 µM) treatment (48 h) on mitochondria and nuclei morphology in Detroit 562 cells. The white arrows show apoptotic-like nuclear changes and mitochondrial alterations. Images were obtained at 20× magnification, and the scale bar was 100 µm.

The mitochondrial damage revealed by the loss of membrane potential following SIL treatment was further assessed by performing mitochondrial morphology staining. In addition, the nuclei were also stained using Hoechst 33342 dye. Microscopic analysis of nuclear and mitochondrial morphology in Detroit 562 cells (Figure 5) treated with SIL (25, 50, 75, 100, and 200 µM) revealed dose-dependent structural alterations. At 25 µM, the nuclei and mitochondria exhibited a morphology comparable to the control group, characterized by a diffuse and uniform fluorescence. The mitochondrial network maintained its reticular organization, with elongated mitochondria and only minimal increases in fluorescence intensity, while the nuclei displayed a regular shape similar to that of untreated cells. At 50 µM, early signs of chromatin condensation and a moderate increase in fluorescence intensity were observed. At 75 and 100 µM, these morphological changes became more evident, marked by stronger fluorescence signals at both the nuclear and mitochondrial levels. Mitochondria showed punctate fluorescent condensations, indicative of fragmentation and disruption of the normal reticular structure. At the highest concentration, 200 µM, pronounced alterations in both nuclei and mitochondria were evident, including loss of cellular integrity, nuclear fragmentation, and disruption of the mitochondrial network, accompanied by an uneven fluorescence distribution.

### 3.6. SIL Treatment Triggers Caspase 9 and Caspase 3/7 Activation

**Figure 6:**
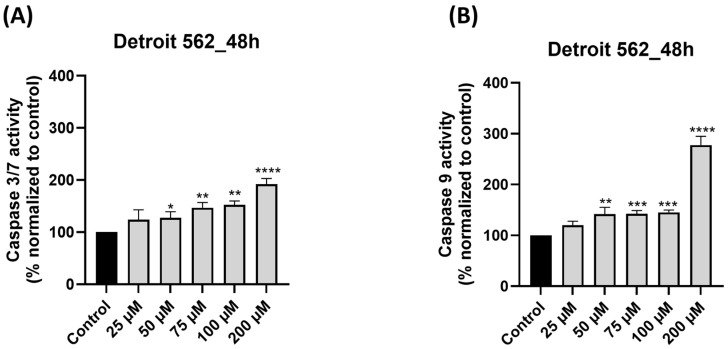
Impact of silibinin (SIL—25, 50, 75, 100 and 200 µM) treatment on: (**A**) caspase 3/7 and (**B**) caspase 9 activity after 48 h of treatment in Detroit 562 cells. The results are expressed as percentages normalized to control and are presented as mean values ± standard deviation. Statistical analysis and definitions of symbols are described in the Statistical analysis section. Data results that were statistically significant were marked with “*”: * *p* < 0.05; ** *p* < 0.01; *** *p* < 0.001; **** *p* < 0.0001.

To further elucidate the mechanism of SIL-induced apoptosis, we analyzed the activation of key intrinsic pathway caspases, namely, caspase-9 and the executioner caspases-3/7. Detroit 562 cells treated with SIL for 48 h showed a dose-dependent elevation of both caspase-3/7 (Figure 6A) and caspase-9 (Figure 6B) activity. Compared with the untreated control (set at 100%), caspase-3/7 activity increased to 124.25%, 127.25%, 146.99%, 152.57%, and 192% at 25, 50, 75, 100, and 200 µM, respectively. Regarding caspase-9 activity, a slight increase was observed at 25, 50, 75, and 100 µM with the following values: 119.78%, 141.84%, 142.39%, and 145.28%. The maximal elevation for caspase-9 level was observed at 200 µM, being 277.53%.

### 3.7. SIL Exhibits a Pro-Apoptotic Effect in Detroit 562 Cells

**Figure 7:**
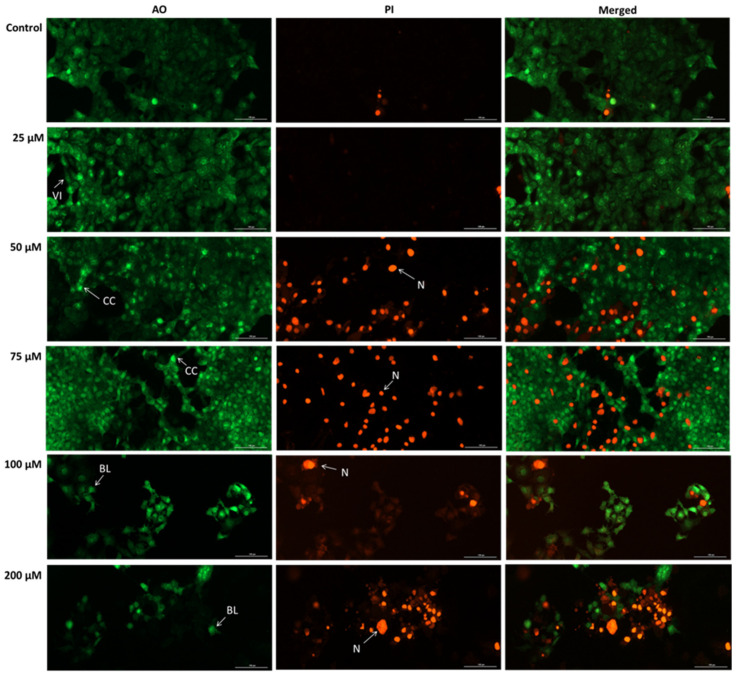
Representative images of the impact of silibinin (SIL 25, 50, 75, 100, and 200 µM) after 48 h in Detroit 562 cells stained with AO/PI. Images were taken at 20× magnification, and the scale bar indicates 100 µm. VI—Viable cells, CC—chromatin condensation, BL—blebbing, N—necrosis.

To confirm the SIL-induced apoptosis in Detroit 562 cells, we conducted a double staining using an acridine orange/propidium iodide (AO/PI) assay. The results showed that SIL treatment induced an increase in apoptotic and necrotic cell death, as indicated by the intensification of the PI (red) fluorescence and the reduction in the AO (green) signal (Figure 7). Viable cells exhibited a diffuse and uniform green fluorescence, which was the predominant appearance of cells treated with SIL at 25 µM. After exposure to 50 and 75 µM beside live cells, early apoptotic cells were identified by a green, fluorescent nucleus displaying chromatin condensation, whereas late apoptotic cells exhibited intense PI-positive red nuclear staining with preserved cell morphology. Beginning at 100 µM, and becoming more pronounced at 200 µM, cells displayed marked cell swelling and diffuse red fluorescence, indicative of loss of membrane integrity, blebbing and secondary necrosis.

### 3.8. SIL Impairs Dose-Dependent the Clonogenic Potential of Detroit 562 Cancer Cells

**Figure 8:**
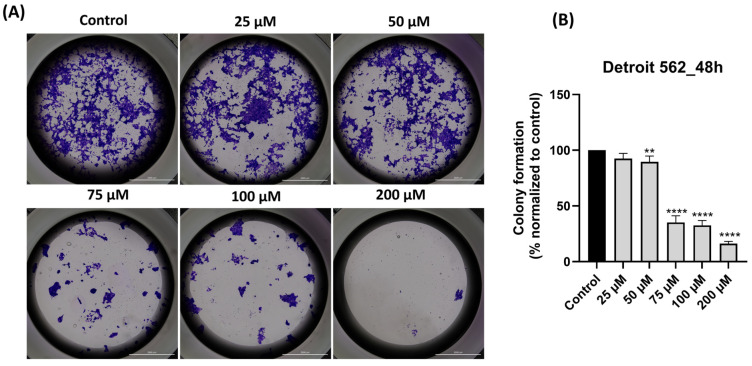
(**A**) Representative images depicting colony formation following 48 h treatment with SIL (25–200 µM) in Detroit 562 cells. Images were taken at 20× magnification, and the scale bar indicates 2000 µm. (**B**) Quantification of colony formation after treatment with SIL for 48 h. The results are shown as a percentage normalized to control and are presented as mean values ± standard deviation. Statistical analysis and definitions of symbols are described in the Statistical analysis section. Data results that were statistically significant were marked with “*”: ** *p* < 0.01 and **** *p* < 0.0001.

The final test performed was to verify the effect of SIL on Detroit 562 cells’ capacity to form colonies. The ability of Detroit 562 cells to form colonies/to proliferate (Figure 8A) was reduced by the 48 h treatment with SIL in a concentration-dependent manner. The percentages of decreased capacity of colony formation normalized to control (Figure 8B) were as follows: 25 µM: 93.77%, 50 µM: 89.50%, 75 µM: 35.18%, 100 µM: 32.53%, and 200 µM: 16.17%. These findings demonstrate that SIL impairs the clonogenic potential of Detroit 562 cells, with the highest concentration producing the most pronounced reduction in proliferation.

## 4. Discussion

In summary, the key findings of this work are: (i) SIL showed a cell-type dependent cytotoxic profile: a potent dose-dependent cytotoxicity in Detroit 562 cancer cells and minimal toxicity in HGF-1 healthy gingival fibroblasts (Figure 1), (ii) SIL altered in a concentration-dependent manner Detroit 562 cells cellular morphology leading to a reduced confluence and apoptotic-like features (rounded cells and shrinkage) (Figure 2), (iii) SIL induced oxidative stress, mitochondrial dysfunction and nuclear alterations (Hoechst 33342), manifested by a gradual increase in intracellular ROS production and loss of mitochondrial potential reported by the JC-1 test (Figure 3, Figure 4 and Figure 5), (iv) SIL triggered caspase 9 and caspases 3/7 activation and induction of apoptosis (confirmed by AO/PI staining) in Detroit 562 cancer cells (Figure 6 and Figure 7), and (v) SIL proved an antiproliferative effect by impairing the clonogenic potential of Detroit 562 cells (Figure 8). Collectively, these results demonstrate that SIL induces apoptosis in Detroit 562 pharyngeal cancer cells via the intrinsic mitochondrial pathway.

HPV-positive and HPV-negative OPSCC dramatically affects quality of life, with high morbidity and mortality rates. In addition to classic risk factors such as tobacco, alcohol, and HPV infection, other critical risk factors include chronic dental trauma, microbiome abnormalities, genetic disorders, and marijuana consumption. In terms of diagnosis, dentists and oral pathologists play a primary role in early detection and diagnosis, with dentists often being the first to identify lesions during physical examinations of the oral cavity [30]. Although HPV-positive OPSCC is the most prevalent form globally, the HPV-negative subtype is predominant in Romania [20,21]. Outcomes for HPV-negative OPSCC are characterized by low survival, with a 5-year overall survival (OS) reported in the range of 27% to 38%. However, the molecular insights of this aggressive phenotype are poorly understood due to a lack of focused studies, highlighting an urgent need for further research [31].

The currently available therapeutic alternatives for OPSCC face multiple obstacles caused by toxicity, complications, and side effects, which need to be overcome or reduced. Consequently, exploring new strategic approaches to improve patient outcomes is imperative, and incorporating natural compounds as potential alternatives is a growing trend of interest [32]. Featuring excellent safety profiles and low toxicity, phytochemicals are considered a desirable area of research for chemoprevention, including for head and neck cancers. Although at an early stage, ongoing studies have already demonstrated the therapeutic utility that natural compounds may serve [33].

The prevalence of HPV-negative OPSCC in Romania and the underexplored treatment strategies for this subtype represented the pillars in designing the present study. SIL (silibinin), a natural compound, was selected as the test compound based on its broad biological profile and its efficacy as an anticancer agent in different cancer types [34,35]. The range of SIL concentrations tested, respectively, 25–200 µM, was chosen based on the scientific literature, which stated that SIL is non-cytotoxic and non-genotoxic at a concentration of 100 µM. Furthermore, Shi-Bing Zhang and colleagues reported that the dose range of 25–200 µM did not cause cytotoxicity in Beas-2B bronchial epithelial cells (noncancerous), nevertheless showing a promising effect on tumor cells [36].

The cell lines serving as 2D experimental models for the present investigation were Detroit 562 pharyngeal human cancer cells and HGF-1 healthy gingival fibroblasts. The Detroit 562 cell line is widely used in scientific research and was chosen for its representation of a pharyngeal squamous cell carcinoma with a p53 gain-of-function mutation due to an R175H amino acid substitution [37]. Mutations in p53 are also common in HPV-negative OPSCC [38]. In addition, Detroit 562 cells have also been previously employed as a preclinical experimental model in other studies targeting various natural compounds as alternatives due to their potential implications in general medicine or dentistry [4,39]. The selection of HGF-1 human gingival fibroblasts as a normal, non-malignant cell line to serve as an experimental model in the present study was based on the following arguments: (i) HGF-1 cells were effectively employed in previous studies conducted on cancer cell lines from the oral/pharyngeal area as an important model for exploring a comparative approach to the differential response of the applied treatments in terms of selectivity for different compounds [40,41,42]; (ii) gingival fibroblasts and pharyngeal cells constitute components of the same anatomical compartment and share common exposure to the same risk factors, such as smoking and alcohol consumption [43]; and (iii) gingival fibroblasts are stromal cells derived from the neural crest, that following activation can transform into cancer-associated fibroblasts that exert crucial roles in driving the progression and metastasis in head and neck cancers, including oral and pharyngeal carcinomas, thereby being in direct connection with the tumor cells [44,45,46].

The primary hypothesis of the present study was that SIL exerts an anticancer effect in Detroit 562 pharyngeal cancer cells through a mitochondria-induced apoptosis mechanism. In this respect, the experimental design included (i) cell viability assessment in both cancer (Detroit 562 cells) and healthy (HGF-1 gingival fibroblasts) cells, (ii) analysis of cellular morphology, (iii) evaluation of intracellular ROS level and loss of mitochondrial membrane potential as early events in mitochondrial dysfunction, (iv) confirmation of mitochondria morphology alterations and nuclear changes, (v) activation of caspase 9 and caspases 3/7, as key regulators of intrinsic mitochondria-mediated apoptosis, and (vi) validation of apoptosis occurrence. Additionally, the antiproliferative effect of SIL in Detroit 562 cells was also tested using the colony formation assay.

Our cell viability results indicated a selective dose-dependent cytotoxic effect of SIL in Detroit 562 cells by reducing cells’ viability percentages (Figure 1A) and a minimal toxic effect in HGF-1 cells (only the highest concentration tested—200 µM slightly decreased cell viability—Figure 1B). These results are in line with previous studies that reported SIL cytotoxic effect in SCC-25 human oral squamous carcinoma cells (concentrations up to 100 µM, for 24 h) [18], in two oral squamous carcinoma cell lines, YD10B and Ca9-22 (50–200 µM, for 24, 48, 72, and 96 h) [47] and in head and neck squamous cell carcinoma (HNSCC) cells [48]. To the best of our knowledge, this is the first study that verified the cytotoxicity of SIL in human pharyngeal cancer cells—Detroit 562. Detroit 562 cells were used as an experimental model in other studies that investigated other flavonoids such as hesperidin, quercetin, and rutin, and similar dose-dependent cytotoxic effects were noted (IC_50_ = 58.83 µM for hesperidin, IC_50_ = 73.23 µM for quercetin, and IC_50_ = 60.53 µM for rutin) [39].

The safety and selectivity of SIL towards healthy cells is an important criterion to consider when evaluating its potential as an anticancer agent. In this regard, a study conducted on human tenon fibroblasts (HTF) showed that SIL (50–100 µM) inhibited platelet-derived growth factor-induced proliferation without causing significant cytotoxicity, with the percentage of dead cells remaining below 15%, thereby indicating a good tolerance of HTF cells to these concentrations [49]. Divyambika Catakapatri Venugopal and the research team evaluated the antifibrotic effect of silymarin on arecoline-induced fibrosis in primary human buccal fibroblasts at concentrations up to 200 µM, finding a limited reduction in cell viability (IC_50_ approximately 143 µM) and, secondarily, that silymarin downregulated cancer progression genes involved in malignant transformation [50].

An interesting finding observed in both cell lines was that SIL treatment at the lowest concentration (25 µM) induced cell stimulation followed by a concentration-dependent cytotoxic effect. One possible explanation for this action could be linked to the hormesis effect. Hormesis is a biphasic dose response to an environmental agent characterized by stimulation at low doses and an inhibitory or toxic effect at higher doses. In fact, it is an adaptive response of cells to moderate stress [51]. Similar effects were reported for different flavonoids (e.g., genistein, quercetin, daidzein), in studies involving cell lines derived from the oral region (SCC-25 squamous cell carcinoma of the tongue) [52]. Nevertheless, further studies are needed to confirm this hypothesis.

As an important part of the cytotoxicity evaluation of a compound, microscopic analysis provides important information on cells behavior after treatment, the underlying biological mechanism, and the possible type of cell death involved [53]. The bright-field morphological analysis of Detroit 562 cells following the 48 h treatment with SIL (25–200 µM Figure 2), indicated a dose-dependent decrease in confluence, with particularly marked rounding, massive shrinkage of cells and their detachment from the well plate at concentrations of 100 and 200 µM, specific signs of cytotoxicity. Another study presented a resembling behavior of SIL (0–300 µM) on the morphology of YD10B and Ca9-22 oral cancer cells following treatment for 48 h, a similar trend of concentration-dependent reduction in cell density, signs of cell rounding and contraction, with the most obvious dysmorphologies visualized at a dose of 300 µM [47].

To validate the primary hypothesis of the study that SIL induces mitochondria-induced apoptosis by upregulating intracellular ROS levels and to gather mechanistic insights, our next step was to evaluate the ROS production in response to SIL treatment in Detroit 562 cells. SIL upregulates ROS production in a dose-dependent manner (Figure 3). ROS play crucial roles in apoptosis and tumor progression. An increased level of intracellular ROS suppresses tumor growth by inhibiting the cell cycle and interferes with apoptosis via the JNK signaling pathway [47]. Our data are supported by other studies that reported ROS production following SIL treatment (0–200 µM for 24 h) in YD10B and Ca9-22 cells [42]. Raina et al. suggested that SIL increases ROS levels and induces oxidative stress in SW480 colorectal cancer cells. Furthermore, this effect leads to the dissolution of mitochondrial potential and subsequently to the release of cytochrome c from mitochondria [54]. ROS plays an important role in the development and progression of oral cancer, including OSCC. As well, lifestyle habits aimed at reducing ROS levels can lower the risk of cancer and improve patient outcomes [55].

To continue the examination of the mechanistic succession of SIL action, the JC-1 bioassay was performed, which primarily monitors mitochondrial function by evaluating the mitochondrial membrane potential. The mitochondrial membrane potential (ΔΨm) (MMP) represents a key indicator of mitochondrial function, reflecting the capacity of mitochondria to produce ATP through oxidative phosphorylation, while its loss serves as an important marker for the initiation of apoptotic processes. The JC-1 method is based on the potential-dependent accumulation of the dye in mitochondria. In polarized mitochondria (high ΔΨm), JC-1 forms red fluorescent aggregates, whereas in depolarized mitochondria, it remains in its monomeric form, emitting green fluorescence. Therefore, the red/green fluorescence ratio reflects the mitochondrial functional state [56]. Our results revealed that SIL treatment in Detroit 562 cells showed a gradual (dose-dependent) decrease in MMP (Figure 4A). This decrease highlights the impairment of mitochondrial function and the disruption of the cell’s ability of energy production, a process commonly associated with the initiation of apoptotic mechanisms [57]. The impact of SIL on MMP has also been evaluated in other cancer cell lines: in human glioma cell lines (U87 and U251), SIL (200 µM) induced a time-dependent decrease in the MMP [58], whereas in PC-12 (rat pheochromocytoma) and HepG2 (human hepatocarcinoma) cells a marked decrease in mitochondrial membrane potential was noticed at a concentration of 150 µM, indicating mitochondrial dysfunction [59].

An elevated level of intracellular ROS and a decrease in mitochondrial membrane potential are associated with mitochondrial impairment. Therefore, the next method applied was focused on confirming mitochondrial morphological alterations and nuclear changes induced by SIL treatment in Detroit 562 cancer cells using immunofluorescence techniques. In cancer cells, treatment-induced alterations at the nuclear level can signal changes in chromatin organization and genome stability, providing valuable insight into how therapeutic agents modulate cellular responses [60]. The results after Hoechst 33342 and Mito Tracker staining (Figure 5) indicated that with increasing concentration, signs of cellular stress progressed in a dose-dependent manner, the most significant effects being observed at the highest concentration of SIL—200 µM: a visible decrease in the number of nuclei and mitochondria, nuclear condensation and nuclear shrinkage, and mitochondrial network collapse. In other cancer cell lines, such as PANC-1, BxPC-3, and AsPC-1, SIL treatment (100 µM) was associated with the presence of nuclei with chromatin condensation and the formation of apoptotic bodies [61]. Comparable pro-apoptotic effects of SIL have been reported in oral and head and neck squamous carcinoma models (YD10B and Ca9-22 oral cancer cells), where SIL induces loss of nuclear integrity in parallel with activation of the mitochondrial apoptotic pathway [47]. In head and neck squamous cell carcinomas, including oropharyngeal tumors, mitochondrial dysfunction is frequently observed, characterized by altered oxidative phosphorylation, increased ROS production, and changes in mitochondrial dynamics. These alterations support tumor cell survival [62].

Caspase cascade activation represents the final point in mitochondria-mediated apoptosis, so the study continued with the investigation of SIL’s effect on caspases 9 and 3/7 activation. Caspases are regulators of programmed cell death, and their function is controlled in a complex manner by epigenetic modifications, various molecular interactions, and post-translational changes, thereby mirroring their pivotal role in cellular homeostasis and disease mechanisms. Caspase 9 is found in mitochondria as a procaspase and in the course of apoptosis, is released from the mitochondria into the cytosol, where it is activated and results in the initiation of the apoptotic cascade. Caspases 3 and 7 are executioners of apoptosis [63]. Our results indicated that SIL elicited dose-dependent activation of caspases 9 and 3/7 in Detroit 562 cells (Figure 6A,B), indicating the initiation and execution of apoptosis. These findings mirror those reported in other studies conducted on oral carcinoma cell lines [47].

To confirm SIL-induced apoptosis in Detroit 562 cells, an acridine orange/propidium iodide (AO/PI) double staining assay was performed. This dual-staining approach allows differentiation between viable, apoptotic, and necrotic cells [64]. The evaluation findings (Figure 7), indicated a dose-dependent increase in apoptosis and secondary necrosis, evidenced by chromatin condensation, the appearance of PI-positive nuclei, and cell blebbing. Similarly, Murali et al. also used a closely related technique, acridine orange/ethidium bromide (AO/EB) dual staining, to evaluate the effect of SIL on SCC-25 cells. Their results demonstrated a dose-dependent increase in apoptotic cells [18]. Furthermore, Vakili et al., showed by using the AO/PI staining assay that SIL (100 µg/mL) induced predominantly apoptotic cell death in HepG2 cells, whereas in HUVEC cells, the predominant mode of cell death was necrosis [65].

According to previous studies [66,67,68], cancer cells have the ability to recover even after they have reached key checkpoints of apoptosis such as mitochondrial fragmentation, activation of caspases cascade, chromatin condensation, DNA injury, nuclear fragmentation, cell shrinkage, and the formation of apoptotic bodies, a phenomenon known as anastasis. This process shows the cancer cells survival capacity after the toxic stimuli were removed, and the cells regain their ability to proliferate, migrate and develop metastasis. In addition to our previous results, the impact of SIL treatment was also assessed on Detroit 562 cells proliferation and on their survival ability by conducting a colony formation assay.

The results of the clonogenic test (Figure 8A,B) after 10 days of cell tracking showed that clonogenic potential (reproductive death) of Detroit 562 cells decreased with increasing SIL concentration. The impairment of colony formation capacity and, at the same time, of migration following SIL treatment was accompanied by the suppression of clonogenic properties and the inhibition of colonization, a process described as micrometastasis formation [27]. Studies have reported that SIL can suppress the invasion and migration of oral cancer cells depending on the dose [47]. Also, regarding clonogenicity and colony formation capacity, Deep et al. showed that SIL treatment inhibited proliferation, clonogenicity, and endothelial cell tube formation in hypoxic PCa cells (1% O_2_) [69]. Furthermore, SIL reduced colony formation even in RT4 and T24 cancer cells [15].

Collectively, our findings indicate that SIL exerts a dual mode of action in Detroit 562 cells, promoting mitochondrial-induced apoptosis while concurrently reducing their proliferative capacity.

However, to the best of our knowledge, the literature has not reported the evaluation of SIL as a natural compound for its antitumor potential in Detroit 562 carcinoma cells. Thus, this article represents the first report on the cytotoxic effect of SIL as a therapeutic alternative in human pharyngeal cancer cells, offering novel insights into the anticancer potential of the phytocompound in oral and pharyngeal cancers through a multi-method analysis and mechanistic approach.

These results indicate the compound’s potential for clinical development in OPSCC prevention. It exhibits a dual mechanism of action: providing hepatoprotection against alcohol-induced liver injury (a major risk factor for HPV-negative OPSCC) and exerting direct cytotoxicity on cancer cells. Another clinically meaningful consideration would be the observed selectivity of SIL towards cancer cells that is critically needed in clinical oncology, considering the barriers imposed by conventional therapy through the induction of toxicity in healthy tissue. Furthermore, a promising approach could be the incorporation of SIL into various dental care products or into delivery systems for oral/local application as preventive agent. In addition to the above, future research directions could also include the assessment of pharmacokinetics and bioavailability, as well as the evaluation of synergistic potential with other chemotherapeutics or standard forms of treatment already in practice. These avenues for future development could complement the present findings with significant clinical results.

Nonetheless, the present study has several limitations that should be addressed in future work. Firstly, this study focused on the influence of SIL in Detroit 562 cell lines as an experimental model. Future investigations should include additional pharyngeal cancer cell lines (both HPV-positive and HPV-negative) with varying degrees of differentiation to obtain comparative results. Complementary to these findings, future research lines could also consider other types of experimental models, such as 3D-reconstructed tissues, spheroids, and in vivo models; these approaches would reproduce the complexity of the tumor microenvironment more accurately and enhance the clinical relevance of the results. Secondly, the concentration range tested was limited to 25–200 µM. Future studies should expand this range and perform a more in-depth exploration of the compound’s anticancer mechanism of action.

## 5. Conclusions

Taken together, these findings confirmed the primary hypothesis of the study that SIL exhibits selective anticancer effects in Detroit 562 pharyngeal cancer cells via mitochondria-induced apoptosis and an antiproliferative effect. The SIL’s anticancer mechanism of action included several key events: (i) a significant decrease in cell viability accompanied by distinct morphological alterations, (ii) stimulation of intracellular ROS, leading to mitochondrial dysfunction and a loss of mitochondrial membrane potential, (iii) activation of apoptotic cascade by the mitochondrial impairment, and activation of caspase 9 and the executioner caspases 3/7, and (iv) reduction in clonogenic potential. These findings open future research directions for the investigation of silibinin (SIL) as a potential candidate for OPSCC therapy, with particular regard to HPV-negative OPSCC.

## Data Availability

The original contributions presented in the study are included. Further inquiries can be directed to the corresponding authors.
